# A framework for identifying cost efficiencies in malaria routine data quality audits: methodology and application in Zambia

**DOI:** 10.1186/s12936-026-05900-5

**Published:** 2026-04-21

**Authors:** An Nguyen, Prudence Malama, Christopher Lungu, Mercy Mwanza, Dingase Phiri, Kafula Silumbe, Japhet Chiwaula, Michael Hainsworth, Smita Das, Arantxa Roca-Feltrer

**Affiliations:** 1 PATH, Ho Chi Minh City, Vietnam; 2 PATH Malaria Control and Elimination Partnership in Africa (MACEPA), Lusaka, Zambia; 3 National Malaria Elimination Centre, Lusaka, Zambia; 4 PATH, Seattle, United States; 5 PATH, Maputo, Mozambique

**Keywords:** Cost efficiency, Health financing, Routine data quality audits, Zambia

## Abstract

**Background:**

Routine data quality audits (RDQA) are a critical activity conducted by many country malaria programs to assess the reliability of reported malaria data, but it requires significant human and financial resources. In collaboration with the Zambia National Malaria Elimination Centre, PATH’s Malaria Control and Elimination Partnership in Africa (MACEPA) proposed a framework for identifying cost efficiencies in RDQAs. To demonstrate this approach, previously collected RDQA data from Zambia was used as a case study.

**Methods:**

A systematic, replicable framework to identify cost efficiencies in malaria RDQA interventions was developed by applying health economic evaluation principles and micro-costing methods. The framework consists of four sequential stages: (1) Conduct a costing analysis to estimate costs of the RDQA interventions by estimating financial and economic costs of all RDQA phases (planning, orientation, audit, feedback); (2) Identify cost drivers of RDQA interventions through comparative analysis of total costs, cost per health facility catchment area (HFCA), and proportional contribution of each cost category, activity, and phase across multiple RDQA interventions; (3) Develop resource-optimized scenarios by adopting the lowest-cost feasible practices; (4) Evaluation of cost-efficiency of scenarios using incremental cost per additional HFCA audited compared to the base-case implementation. Nine RDQA interventions implemented by four projects in Zambia (2022–2024) covering 10 provinces, 91 districts, and 1,189 HFCAs were evaluated.

**Results:**

The proposed framework estimated the average economic costs of $24,938 per intervention ($205 per HFCA), with personnel (64%) and transportation (25%) as primary cost drivers of RDQAs in Zambia. Optimized scenarios incorporating virtual orientation meetings, streamlined audit team composition, and harmonized allowances reduced total costs by 41%. Transition to digital RDQA tools projected additional savings of up to 52%.

**Conclusion:**

This structured four-stage framework provides a practical methodology for identifying substantial cost efficiencies in RDQA interventions while maintaining or expanding coverage and quality. The analysis has strong implications for policy makers, funders and implementers of malaria programs on optimizing resource utilization for maintaining high-quality routine malaria data. The approach can extend to other disease-specific interventions or analogous activities, such as supportive supervision visits, enabling more sustainable implementation in countries facing similar resource constraints in health programs.

**Supplementary Information:**

The online version contains supplementary material available at 10.1186/s12936-026-05900-5.

## Background

A malaria Routine Data Quality Audit (RDQA) is the process of assessing accuracy of reported malaria surveillance data at the facility level, identifying and addressing challenges with data recording, and making corrections to have precise data reported to intermediate and national levels. RDQAs are critical for ensuring accurate and reliable use of routine data for decision making [1–4]. Nonetheless, the process itself has been facing challenges of high costs, quality of implementation, and deprioritized investments [1, 5–7]. As the global funding for malaria has increasingly fallen short to meet the Global Technical Strategy (GTS) target over the last five years, with only 43% of the funding target met in 2023 and an expectation that this gap will continue, there are concerns that funding shortages will affect budgets for data quality assurance activities [7, 8]. Due to limited human and financial resources available to conduct RDQAs, National Malaria Control Programs (NMCP) face significant challenges in implementing wide-scale data quality improvement activities. Variations in RDQA approaches, frequency, and scope create diverse implementation models and budget needs.

However, no formal review of RDQA costs has been conducted to guide the optimization of resources. Therefore, there is a need to review and document RDQA-associated costs to establish possible benchmarks of the expenses and identify potential alternative solutions to save costs. PATH’s Malaria Control and Elimination Partnership in Africa (MACEPA) [9], in close collaboration with the Zambia National Malaria Elimination Centre (NMEC), developed a framework for identifying cost efficiencies in malaria RDQAs and used RDQA data in Zambia as a case study to identify key cost drivers and to make recommendations for less costly implementation to achieve similar outcomes. The approach is primarily aimed at being used for malaria RDQAs but can be applied to other disease programs.

## Methods

### An overview of the approach

Standard costing methods, principles of economic evaluations of health care programs, costing framework for malaria interventions, and experiences of implementing partners [10–19] were used to develop an approach for identifying cost efficiencies in RDQA implementation. Specifically, the approach includes the four following stages to identify cost efficiencies and make recommendations for RDQA interventions:

1. Conduct a costing analysis to estimate costs of RDQA interventions by estimating financial and economic costs of all RDQA phases;
2. Identify cost drivers of RDQA interventions through comparative analysis of total costs, cost per health facility catchment area (HFCA), and proportional contribution of each cost category, activity, and phase across multiple RDQA interventions;
3. Develop resource-optimized scenarios by adopting the lowest-cost feasible practices (validated by RDQA experts);
4. Evaluation of cost-efficiency of scenarios using the cost-effectiveness ratio where incremental cost per additional HFCA audited compared to the base-case implementation [15, 16].

### A costing analysis

It is necessary to identify the costs associated with RDQAs prior to exploring cost-efficiencies. A costing analysis was conducted to estimate the costs of RDQA interventions using a micro-costing approach and a published framework for evaluating the costs of malaria elimination interventions and surveillance systems in Ethiopia, Senegal, and Zambia [11, 13, 20]. The costing analysis included information gathering and analysis to estimate unit costs, and then combined that data with RDQA implementation data at the geographic unit of analysis (i.e. HFCA) to create the cost database. The approach used a bottom-up, ingredient-based method which identified all cost categories and cost items in each category for all activities. The quantity of each item and its unit cost were multiplied and added together to determine the total cost of the intervention.

Applying this framework, the RDQA costing analysis was conducted by first gathering RDQA implementation information by different interventions and identifying main RDQA activities, cost categories and cost items in each category.

#### RDQA implementation by different interventions

**Fig. 1:**
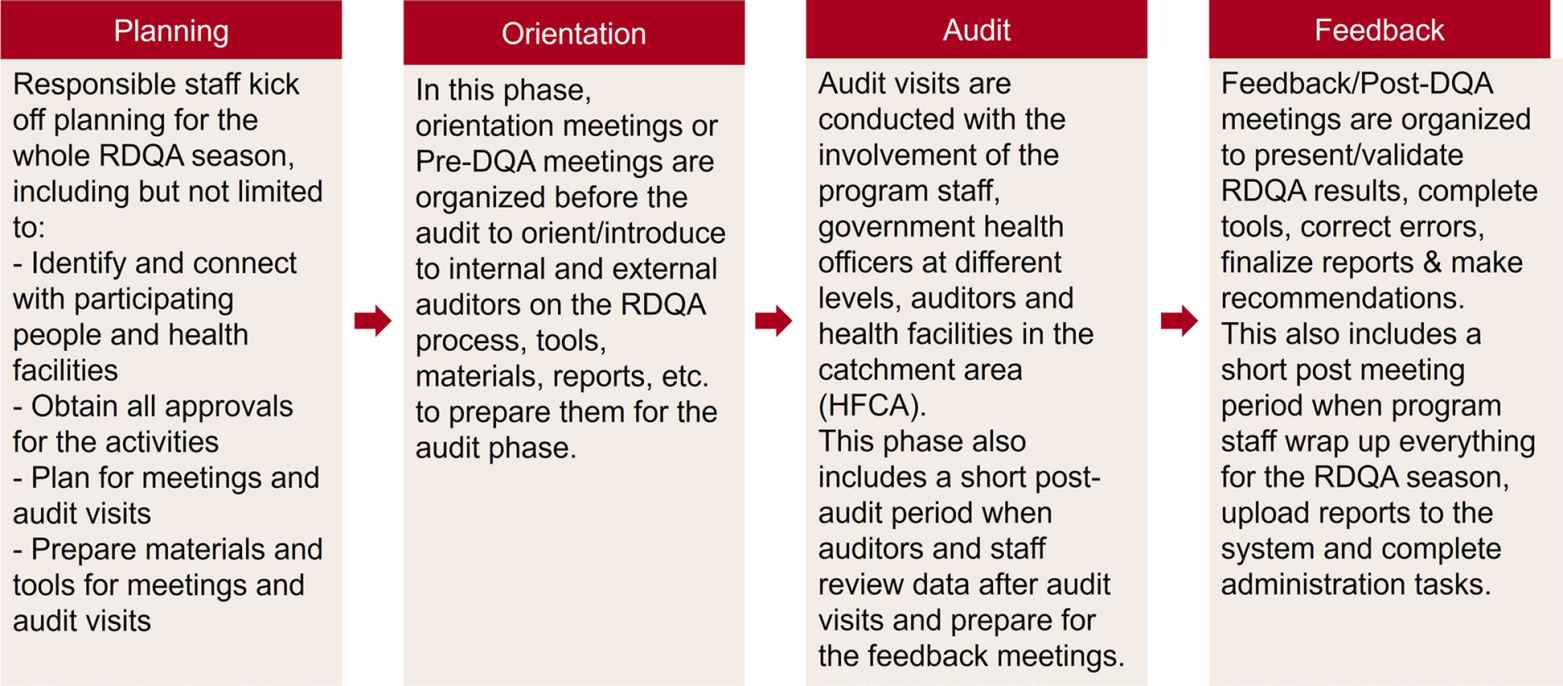
Four phases of RDQA implementation

Each RDQA implementation intervention was characterized by geographic units and catchment areas, the audit process from planning phase to pre-audit, audit and post-audit phases, the activities in each phase (e.g. training, meetings, site visits, etc.), and the entities and number of staff involved in the process. Figure 1 provides the overview of four phases of the RDQA process and main activities in each phase. The RDQA interventions received funding from donors to conduct audits in different areas (e.g. provinces) at different times. Each RDQA intervention spanned a time horizon of up to 12 months.

#### Cost perspectives

Costs incorporated incremental costs from the providers’ perspective, including RDQA implementers (i.e. Ministry of Health, NMCP, development organizations, local and international NGOs, different levels of the health system from national to HFCAs). Both financial costs and economic costs were evaluated. Financial costs included actual expenses incurred for the interventions, while economic costs included aforementioned financial costs and opportunity costs. Depending on data availability, opportunity costs may include previous capital purchases, in-kind contributions, and labor provided by individuals involved in RDQAs with or without allowances.

#### Cost categories and unit cost database

Resources needed for RDQA implementation fell into the cost categories as included in Table 1.

**Table 1:** Cost categories and cost items of RDQA implementation

Cost categories	Cost items and estimation description
Personnel	Included working time, daily wages, and allowances for different positions involved in the process •*Working time* individual’s time spent on planning phase, audits, preparation and wrap-up periods (post-audit visits and post-feedback meetings) •*Wages* Due to confidentiality, wages were not specific to any individual, but rather the average rates for different groups including supporting program staff, government staff, and other staff •*Allowances* varied by RDQA interventions, staff levels and locations
Transportation	Included transport costs for program-owned and rented vehicles and transport allowances •Costs for program-owned and government vehicles used during the RDQA process: fuel costs multiplied by travel distance, tolls and fees, and vehicle maintenance costs during RDQA implementation •Costs for rented vehicles including cars, boats, ox carts for difficult terrains cover costs for renting vehicles, drivers, fuel, and tax
Equipment	Included any equipment required for RDQA activities that were purchased and used for several years such as laptop, printer, cell phone, etc. (also referred to as fixed, non-recurrent or capital costs) The one-time payment for equipment was converted to an annualized equivalent cost using the expected equipment lifespan and a discount rate of 3% (specific for Zambia case study)
Supplies	Included variable or recurrent costs of supplies for RDQA training, meetings, and audit visits such as stationery and hygiene supplies (face masks, hand sanitizers, etc.)
Meals and accommodation	Included accommodation for individuals, venues for meetings, meals and refreshments for training, meetings, and audits. Accommodation was usually for program staff, government staff, and external auditors who travelled to a different province or a distant district for the meetings, trainings, and audits
Others	Any other costs not listed above if any (such as costs for media, photographs)

Unit costs and quantity of cost items were collected from RDQA interventions and used to calculate costs by cost categories and RDQA activities.

#### Activity-specific costs and RDQA intervention costs

Costs in different categories were summed up for activities (training, meeting, visit, etc.) in each phase of the process (i.e. planning, orientation, audit, feedback) based on RDQA implementation information by each intervention. The sum of all ingredient RDQA activities in all phases results in the total RDQA implementation costs by different supporting programs.

### Cost drivers of RDQA interventions

Costs of different RDQA interventions were described in terms of total costs and costs per health facility catchment area (HFCA). The proportions of cost for each cost category, each RDQA activity, and each phase to the total costs were examined to identify the cost drivers across RDQA interventions. Costs of different interventions conducted in different years were converted to 2025 costs using the country’s consumer price index data [21].

RDQA implementation practice and conduct are described to characterize the cost drivers of RDQA interventions with highest and lowest cost or cost per HFCA. This step is important to develop resources-optimized scenarios and make recommendations for cost-efficient RDQA implementation.

### Development of resources-optimized scenarios

For each phase of RDQA implementation (planning, orientation, audit, feedback) and for each cost category (personnel, transport, equipment/supplies, accommodation, others), the practice or implementation approach of the RDQA intervention with the lowest cost per HFCA was identified. Then, RDQA experts were consulted to understand the feasibility of the low-cost practice or implementation approach as an alternative for future RDQA interventions. If the proposed RDQA approach is considered feasible in that particular context, the RDQA approach is included in the resources-optimized scenario(s). For example, the orientation meeting cost in the orientation phase was lowest when conducted virtually. RDQA experts agreed that virtual orientation meetings would achieve meeting objectives without compromising quality compared to current practice (1). Virtual orientation meetings were then included in the resources-optimized scenario(s).

There were specific factors or combinations of factors that did not give a clear picture of cost savings for RDQAs. In these cases, different scenarios were developed by varying the factors or the combination of factors to compare their cost-efficiencies using a sensitivity analysis, which is referred to as a scenario analysis in this paper. For example, increasing the number of audited health facilities would increase total costs, but it was unclear if increasing the number of audited districts and reducing the number of audited health facilities per district or vice versa would be more cost-saving. As a result, scenarios with different combinations of number of districts and HFCAs were compared to identify which scenario is the most cost-efficient.

### Identifying cost-efficient RDQA scenarios

We used the incremental cost-effectiveness ratio (ICER), a metric comparing the cost and outcomes of different health interventions to assess the cost-efficiency of each RDQA scenario [10, 15, 16]. We calculated the difference in total costs of the resources-optimized RDQA scenario against current RDQA implementation (base case) in relation to the difference in number of audited HFCAs in these two comparing scenarios, as shown in the formula below:

$$ICER= \frac{{Cost}_{scenario A }- {Cost}_{base case }}{{\# HFCA}_{scenario A }- {\# HFCA}_{base case}}$$

The lower the ICER, the more cost-efficient the resources-optimized scenario for conducting RDQAs. For scenarios in which the number of audited HFCAs is indifferent from the base case, scenarios with lower costs are considered more cost-efficient. Characteristics of the most cost-efficient scenarios were used to make recommendations for RDQA interventions.

### A case study of RDQA interventions in Zambia

To demonstrate the application of this framework, we conducted a case study to identify cost efficiencies for RDQA interventions implemented in Zambia from 2022 to 2024. Administrative data was collected between November 2024 and January 2025 for nine RDQA interventions conducted by programs led by the GRZ and implementing partners, including the Malaria Control and Elimination Partnership in Africa (MACEPA) funded by the Bill & Melinda Gates Foundation (BMGF), the Program for the Advancement of Malaria Outcomes (PAMO) funded by the President’s Malaria Initiative (PMI), Global Fund (GF), Churches Health Association of Zambia (CHAZ) funded by GF. The RDQA costing analysis was conducted for nine RDQA interventions using their records on unit costs and quantity of cost items for all activities in the four phases of RDQA implementation. All RDQA costs were converted to 2025 USD using the Zambia consumer price index [21] and an exchange rate of 1 USD = 25.39 ZMW.

Costs of these nine RDQA interventions were calculated in total costs and costs per HFCA, along with the characteristics of each intervention for identifying the cost efficiency. The practice and conduct of the RDQA implementation across nine interventions that had the lowest cost or cost per HFCA were then characterized. With consultancy from the RDQA experts, these characteristics were used to construct resource-optimized RDQA scenarios to estimate the impact on cost saving and provide recommendations on cost-efficiencies for RDQAs in Zambia. In these scenarios, we unified the allowances for staff with the same position and level across the interventions but used the local price for unit costs of other items. A new electronic RDQA approach using digital tools is being planned for introduction in Zambia in late 2025 and was also assessed in the scenarios.

## Results

### Costs of RDQA interventions

The costing analysis covered nine RDQA interventions conducted by the GRZ and implementing partners in ten provinces encompassing 91 districts and 1189 HFCAs in Zambia from 2022 to 2024 (Table 2).

**Table 2:** General characteristics of RDQA implementations in the analysis

RDQA intervention	Provinces	# Districts/# HFs audit	# HF staff in audit	O/F meetings format	Refund scheme
CHAZ	North Western	3/42	126	Multiple meetings conducted at each district at the same time	Fuel refund for GRZ vehicles in O/A/F
GF	Western & Central	8/80	102	One meeting for both provinces, virtually or in-person	Fuel refund for GRZ vehicles in A only, transport refund for external auditors
MACEPA 1	Southern & Central	17/173	205	One meeting for both provinces, in-person	Fuel and transport refund for O/F, not for A
MACEPA 2	Western	16/160	183	Meeting at province	Fuel and transport refund for O/A/F
PAMO 1	Eastern Main	8/123	200	Meeting at province	Fuel and transport refund for O/A/F
PAMO 2	Eastern Pre-elimination	7/162	415	Meeting at province	Fuel and transport refund for O/A/F
PAMO 3	Luapula	12/167	200	Meeting at province	Fuel and transport refund for O/A/F
PAMO 4	Muchinga	8/114	80	Meeting at province	Fuel and transport refund for O/A/F
PAMO 5	Northern	12/168	150	Meeting at province	Fuel and transport refund for O/A/F

*HF* health facilities, *GRZ* Government of Republic of Zambia, *O/A/F* orientation, audit, feedback phases

**Fig. 2:**
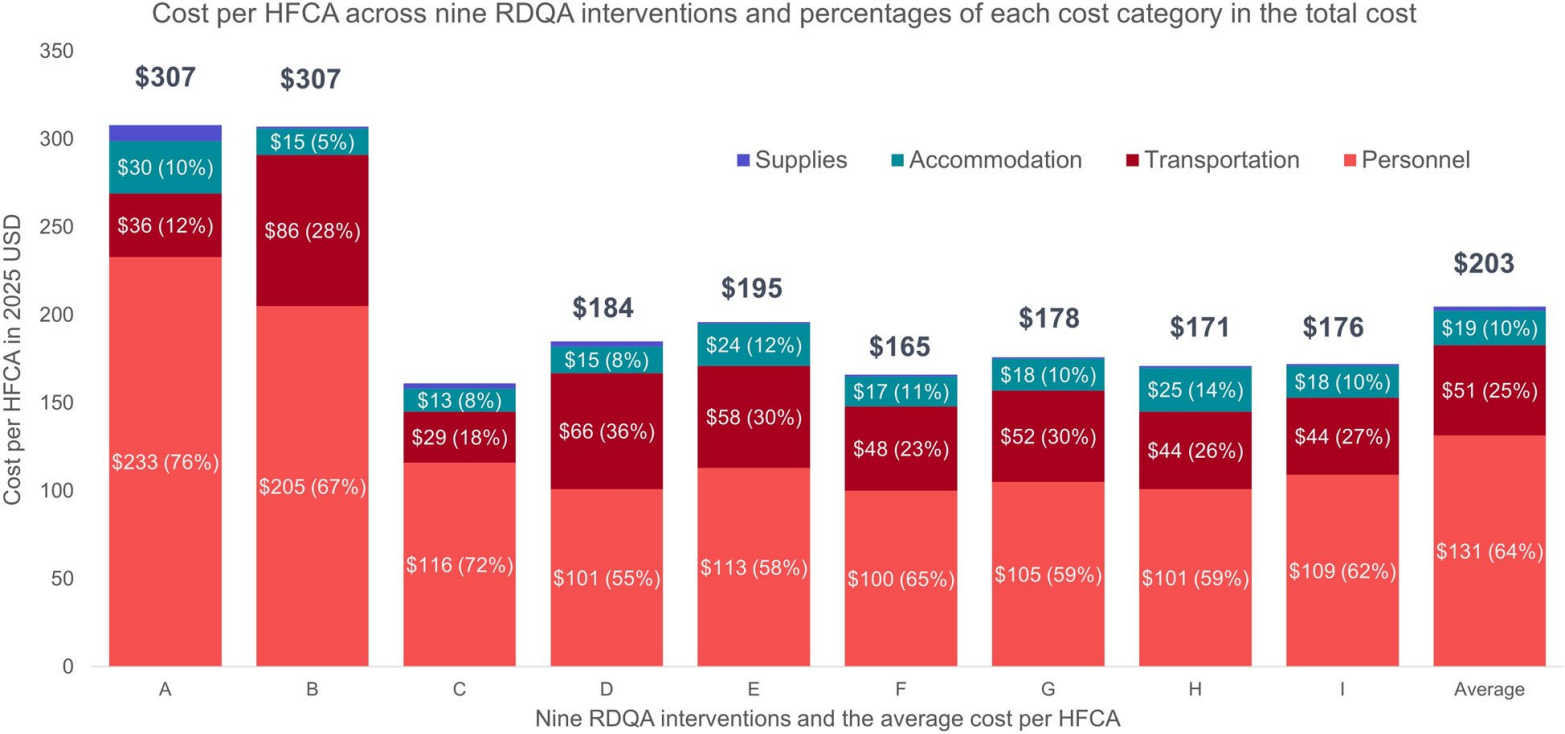
Cost per HFCA and cost drivers of RDQA implementations

There were substantial variations in RDQA costs across the interventions (Fig. 2), with the average economic costs of $24,938 per intervention (range: $12,893–$29,654) and $205 per HFCA (range: $161–$307). The financial costs, which excluded opportunity costs for staff time, averaged $23,293 per intervention and $190 per HFCA.

### RDQA cost drivers

Personnel costs emerged as the primary cost driver, comprising 64% of total costs on average (range: 55–76%), followed by transportation costs at 25% (range: 12–36%). The number of staff at each level participating in audit and feedback phases, as well as the length of these phases, were determinants of the personnel costs. Fuel and transport refunds for audit phase were determinants for the transportation costs.

### Resource-optimized scenarios

The costing analysis findings demonstrated that conducting orientation meetings virtually was the most economical approach at only $1 per HFCA as compared to $51 per HFCA for organizing district-level orientation meetings. The interventions that organized orientation and feedback meetings centrally at the provincial level rather than separately by districts achieved significant cost savings. While the meetings should be centralized, the audit team should be decentralized to involve more district-level staff (local district and external district) for better cost-efficiency. The resources-optimized scenarios were built to audit around 140 HFCAs, which was the average number of audited HCFAs across the nine interventions.

### Cost efficiencies of RDQA scenarios

**Fig. 3:**
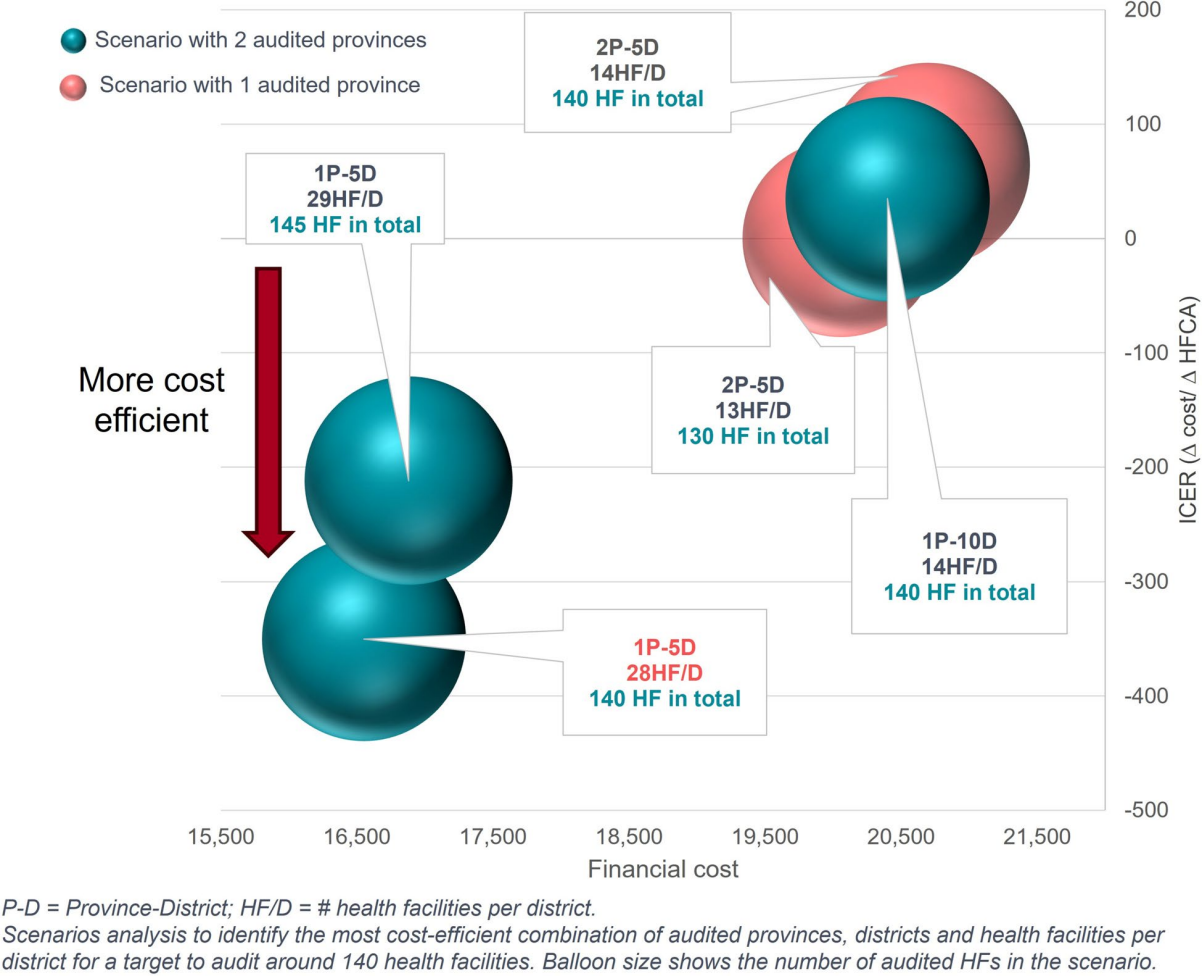
Scenario analysis on the cost-efficient number of provinces, districts, health facilities per district to audit 140 HFCAs

The scenario analysis to determine the most cost-efficient combination of number of provinces, districts, and health facilities per district revealed that reducing the number of districts while increasing HFCAs per district was more cost-efficient than maintaining many districts with fewer HFCAs each, as shown in Fig. 3.

**Fig. 4:**
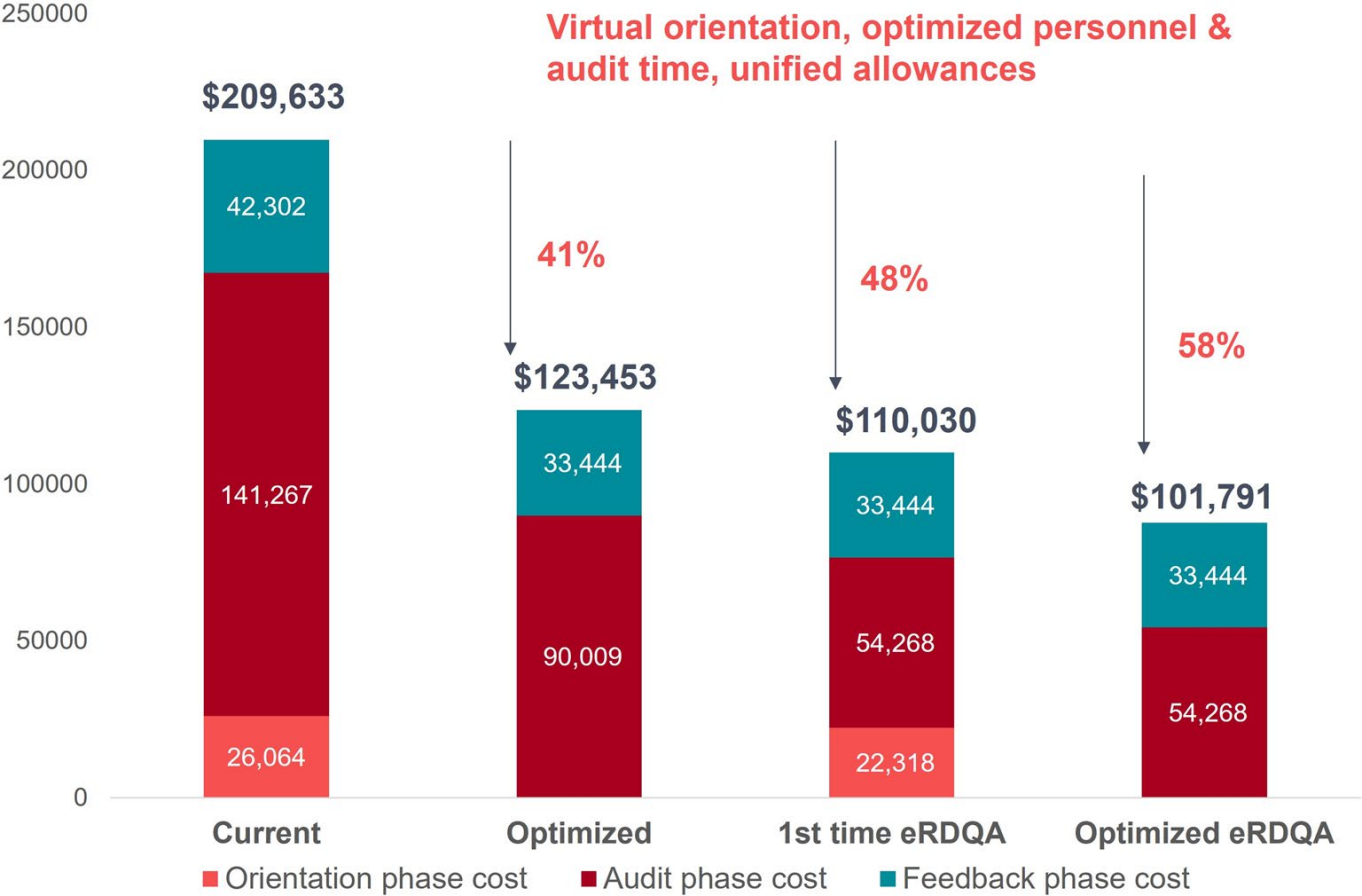
Total financial costs of nine RDQA implementations with current practice vs more cost-efficient practice

The scenario modeling also showed that optimizing current implementation through virtual orientation meetings, streamlined personnel deployment, and unified allowances could reduce the total costs of the nine RDQA interventions by 41% (from $209,633 to $123,453) while maintaining the annual RDQA coverage of 1196 HFCAs. The scenario of using a digitalized electronic RDQA module integrated into DHIS2 [22] as opposed to the traditional more labor-intensive paper and Excel approach, projected even greater potential savings of 52% as compared to current implementation, primarily through the expected reduction of audit time by 50% despite requiring initial training investments (Fig. 4).

## Discussion

Several economic evaluations have been conducted for malaria control interventions but most of them focused on vector control, chemoprevention, diagnostics and treatment. Only about 10% of the studies assessed the costs of surveillance and none of them were about the costs of RDQAs [13, 23–27]. This study provides the first estimations for the costs of RDQA implementation in Zambia and identifies personnel and transportation as the major cost drivers for RDQA process. Personnel costs, specifically salaries, were also identified as major cost-drivers in costing studies on malaria surveillance systems where cost determinants were assessed [28, 29].

By analyzing the variations in costs and conduct of RDQAs across interventions, the approach allows making recommendations for optimizing resources used to implement RDQAs. Based on the findings, we recommend the following strategies for Zambia to maximize RDQA cost efficiency while maintaining quality:

- *Optimized meeting structures* Implement virtual orientation meetings and organize feedback meetings centrally at the provincial level rather than conduct separate district-level meetings.
- *Strategic resource allocation* When facing budget constraints, reduce the number of districts covered while increasing the number of HFCAs audited per district.
- *Standardized audit teams* For medium-volume HFCAs, deploy teams comprising 2 program staff, 2 national officers, 2 provincial officers, 4 local district staff per district, and 2 community health workers per HFCA. While meetings should be centralized, audit teams should remain decentralized to maximize local participation and cost efficiency.
- *Harmonized allowances* Unify allowance rates across different RDQA interventions and consider eliminating transport allowances for local staff during the audit phase to achieve additional cost savings without compromising audit quality.
- *Investment in digitalization* Prioritize the transition to eRDQA systems, which can reduce costs by reducing the amount of time needed to conduct an audit. Consider piloting self-RDQA models and remote auditing options for external auditors to further reduce costs while maintaining data quality standards.

To the best of our knowledge, this is the first cost efficiency evaluation framework developed for malaria RDQAs. The evaluation used the cost data collected from RDQA interventions. Although we examined both financial costs and economic costs for all RDQA implementers including the Ministry of Health, NMCP, supporting organizations, and different levels of the health system from national to HFCAs, we relied on data availability from the programs. The costs of RDQAs could be underestimated as the case study could not obtain granular cost data from the audited health facilities. Furthermore, although the resources-optimized scenarios were developed based on past RDQA implementations, the feasibility of implementing optimized scenarios to other areas and contexts was agreed by RDQA experts and thus should be validated in real practice. Despite the limitations, the framework provides an approach to review RDQA costs and propose ways for more cost-efficient RDQA implementation.

## Conclusion

The framework describes a step-by-step approach to conduct the costing and scenario analysis to identify cost-efficient implementation of malaria RDQAs. The case study using Zambia RDQA data has demonstrated the use of the proposed framework for identifying cost efficiencies in malaria RDQAs and making recommendations for future RDQA interventions in Zambia. The analysis has provided insights into optimizing resource utilization for maintaining high-quality routine malaria data, demonstrating substantial potential for cost savings through strategic efficiencies such as virtual meetings, centralized planning, and digital transitions. While these findings are rooted in Zambia’s context, they offer transferable lessons for other countries facing similar resource constraints in health data management. The approach can extend to other disease-specific interventions or analogous activities, such as supportive supervision visits, enabling more sustainable implementation without compromising quality or coverage.

## Supplementary Information

Supplementary Material 1.

## Data Availability

All data, supporting information and analysis are provided in the additional file “Supplementary-MACEPA-combined.xlsx”.

## Abbreviations

DHIS: District health information software
eRDQA: Electronic routine data quality audits
HFCAs: Health facility catchment areas
MACEPA: Malaria Control and Elimination Partnership in Africa
NGOs: Non-governmental organizations
NMCP: National Malaria Control Programs
NMEC: National Malaria Elimination Centre
RDQAs: Routine data quality audits
